# Promising diagnostic and therapeutic circRNAs for skeletal and chondral disorders

**DOI:** 10.7150/ijbs.57887

**Published:** 2021-04-07

**Authors:** Gaoyang Chen, Wanze Tang, Shang Wang, Canling Long, Xiaoqin He, Dazhi Yang, Songlin Peng

**Affiliations:** Department of Spine Surgery and Institute for Orthopaedic Research, the 2nd Clinical Medical College (Shenzhen People's Hospital) of Jinan University, the First Affiliated Hospital of Southern University of Science and Technology, Shenzhen Key Laboratory of Reconstruction of Sports System, Shenzhen, 518055, China.

**Keywords:** Circular RNAs, skeletal and chondral disorders, regulatory mechanism, biomarker, therapeutic target.

## Abstract

Circular RNAs (circRNAs) belong to a highly conserved subtype of non-coding RNAs, produced by the back-splicing of specific regions of pre-mRNA. CircRNAs have wide-ranging effects on eukaryotic physiology and pathology by acting as transcription regulators, miRNA sponges, protein sponges, and templates for translation. Skeletal and chondral disorders are the leading causes of pain and disability, especially for elders, affecting hundreds of millions of people worldwide. Plenty of evidence have shown that circRNAs are dysregulated and play vital roles in the occurrence and progression of skeletal and chondral disorders. Herein, we systematically summarize the emerging roles and underlying molecular mechanisms of hub circRNAs in the pathogenesis of several representative skeletal and chondral disorders. Our findings may provide further insight into the mechanistic details of the role of circRNA in bone or cartilage metabolism, and highlight the promising application of circRNAs in serving as potential diagnostic or therapeutic targets for the prevention and treatment of skeletal and chondral disorders.

## Introduction

**Figure 1:**
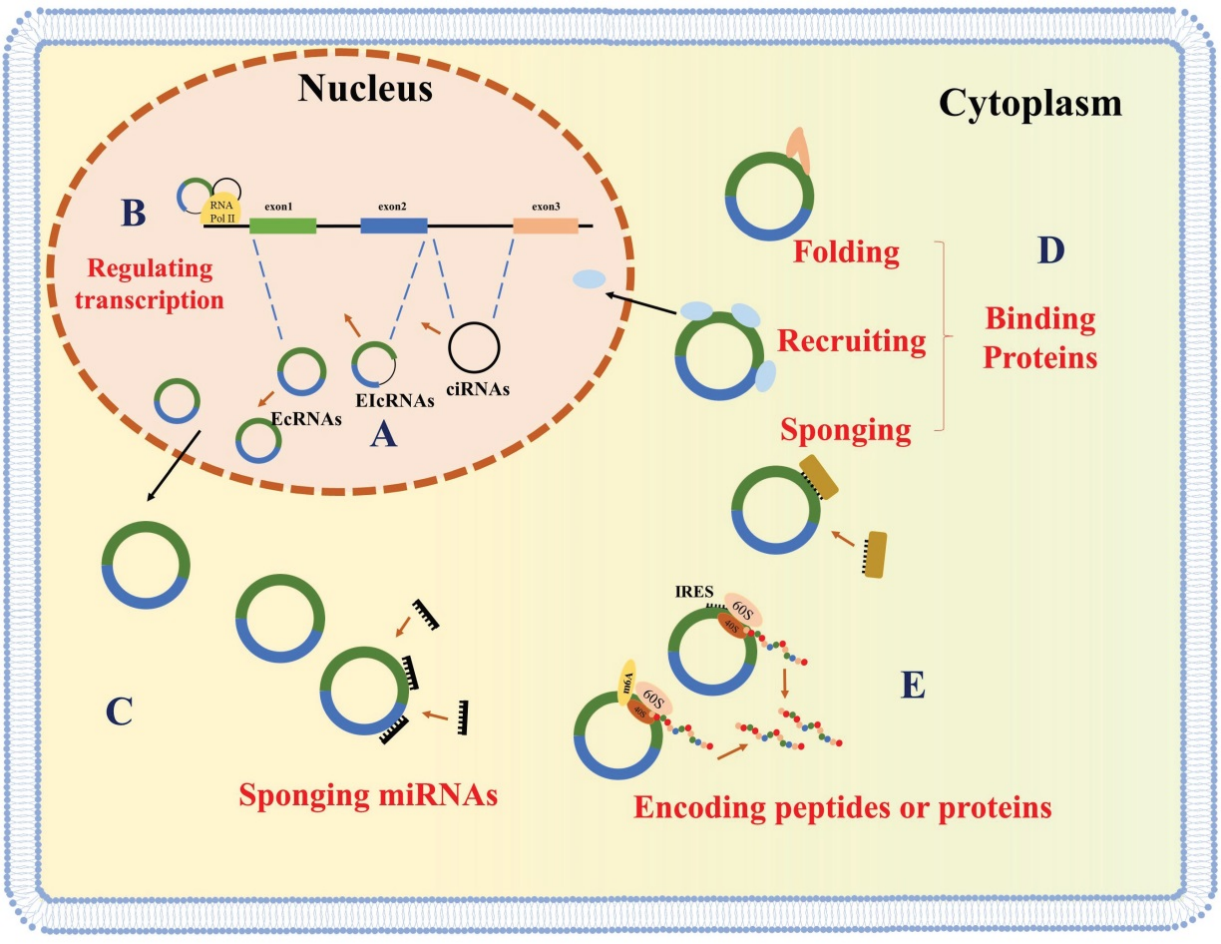
** Biogenesis and molecular functions of circRNAs.** CircRNAs are generated by back-splicing **(A)** and regulate gene expression at the level of transcription **(B)**, sponging miRNAs **(C)**, interacting with proteins **(D)**, or encoding peptides **(E)**.

Circular RNAs (circRNAs) are recently discovered non-coding RNAs (ncRNAs) that consist of a specially covalently closed ring structure and can be stably expressed in multiple cell lines 1, 2. CircRNAs derive from canonical splice sites, upon suppression or slowing down of the splicing of pre-mRNA 3, 4. CircRNAs are classified based on their sequence. There three types of circRNAs are exonic circRNAs (EcRNAs), intronic circRNAs (ciRNAs) and exon-intron circRNAs (EIcRNAs) (**Figure 1A**) 5, 6. EcRNAs are mainly found in the cytoplasm, while ciRNAs and EIcRNAs are predominantly found in the nucleus. EcRNAs are the most abundant circRNAs and account for over 80% of the known circRNAs 1, 7, 8.

CircRNAs were first discovered in pathogens in the 1970s 9 and were initially thought to be transcriptional splicing intermediates or byproducts of pre-mRNA splicing errors 10, 11. Since then, due to the advancements in RNA sequencing technologies and bioinformatic analyses, thousands of circRNAs have been identified 12. Multiple lines of evidence have highlighted the significant impact of circRNAs on various eukaryotic physiological and pathological processes 13. CircRNAs in the nucleus (ciRNAs and EIcRNAs) play roles in host gene transcription regulation (**Figure 1B**) 5, 14, 15, while cytoplasmic circRNAs have been shown to function as miRNA sponges, thereby promoting the expression of downstream mRNAs (**Figure 1C**) 7, 16, 17. Recently, several circRNAs have been implicated in the function and localization of proteins by serving as a decoy, and facilitating protein folding and recruitment (**Figure 1D**) 7, 16-20. Some studies have also shown that coding circRNAs can play critical roles in human diseases (**Figure 1E**) 21-24.

CircRNAs function as pervasive regulators of cellular and physiological processes and have been proven to be vital in many diseases, such as skeletal and chondral disorders 25-27. Skeletal and chondral disorders are among the leading debilitating factors and represent an increasing societal and economic burden in the context of aging population and increasing life expectancy. The most common of these diseases are osteoarthritis, osteonecrosis of the femoral head (ONFH), osteoporosis, and rheumatoid arthritis (RA), affecting hundreds of millions of people worldwide 28-31. Several factors have been associated with the occurrence and progression of skeletal and chondral disorders, including inflammation, apoptosis, degradation of extracellular matrix (ECM), and an imbalance between osteogenesis and adipogenesis 30, 32-36. However, the underlying molecular mechanisms of the pathology of these diseases still remains elusive, and an understanding of these mechanisms is essential for both the prevention and treatment of bone and cartilage disorders. Studies have begun to highlight the differential expression of circRNAs in bone and cartilage diseases as well as their regulatory roles 37. Further characterizing the roles of circRNAs in the pathological processes of skeletal and chondral disorders could provide new avenues for both diagnosis and treatment.

In this review, we summarize recent studies of circRNAs involved in common skeletal and chondral disorders, such as osteoarthritis, ONFH, osteoporosis, and RA, and highlight the potential applications of these circRNAs in the prevention and treatment of these disorders.

## CircRNA and osteoarthritis

Osteoarthritis is recognized as the most common disease of the musculoskeletal system, with up to 250 million cases globally 38. Cartilage damage associated with osteoarthritis can be caused by mechanical, inflammatory, or metabolic stresses that alter cartilage properties 39-41. As a result, hypertrophic chondrocytes attempt to repair eroded cartilage, but in turn release more matrix degradation products and proinflammatory factors, which then leads to structural alterations in articular cartilage, subchondral bone, synovium, capsule, ligaments, and periarticular muscles 42-44. Here, we discuss osteoarthritis-related circRNAs and their regulatory effects on inflammatory and matrix metabolic factors in osteoarthritis-related cells. Understanding the role of these circRNAs can reveal new strategies for intervening the progression of osteoarthritis.

### Diagnostic circRNAs of osteoarthritis

CircRNAs have been widely recognized as stable biomarkers due to their resistance from degradation by RNases 45. The use of diagnostic circRNAs in peripheral blood and synovial fluid could have a significant impact on osteoarthritis diagnosis. Studies have indicated that hsa_circ_0104873, hsa_circ_0104595, and hsa_circ_0101251 are steadily expressed in synovial fluid, while hsa_circ_0032131_CBC1 is significantly over-expressed in the blood samples of osteoarthritis patients (**Table 1**) 46, 47. Further investigation of these circRNAs could set the groundwork for the advancement of minimally invasive, highly specific, easily accessible, and rapid diagnostic biomarkers.

### Regulatory circRNAs of osteoarthritis

Articular cartilage degeneration is the vital process in the pathogenesis of osteoarthritis 48. The imbalance of cell proliferation and apoptosis of chondrocytes finally results in the increased catabolism and decreased anabolism of ECM, thereby leading to the inflammation and degeneration of cartilage 48.

**Figure 2:**
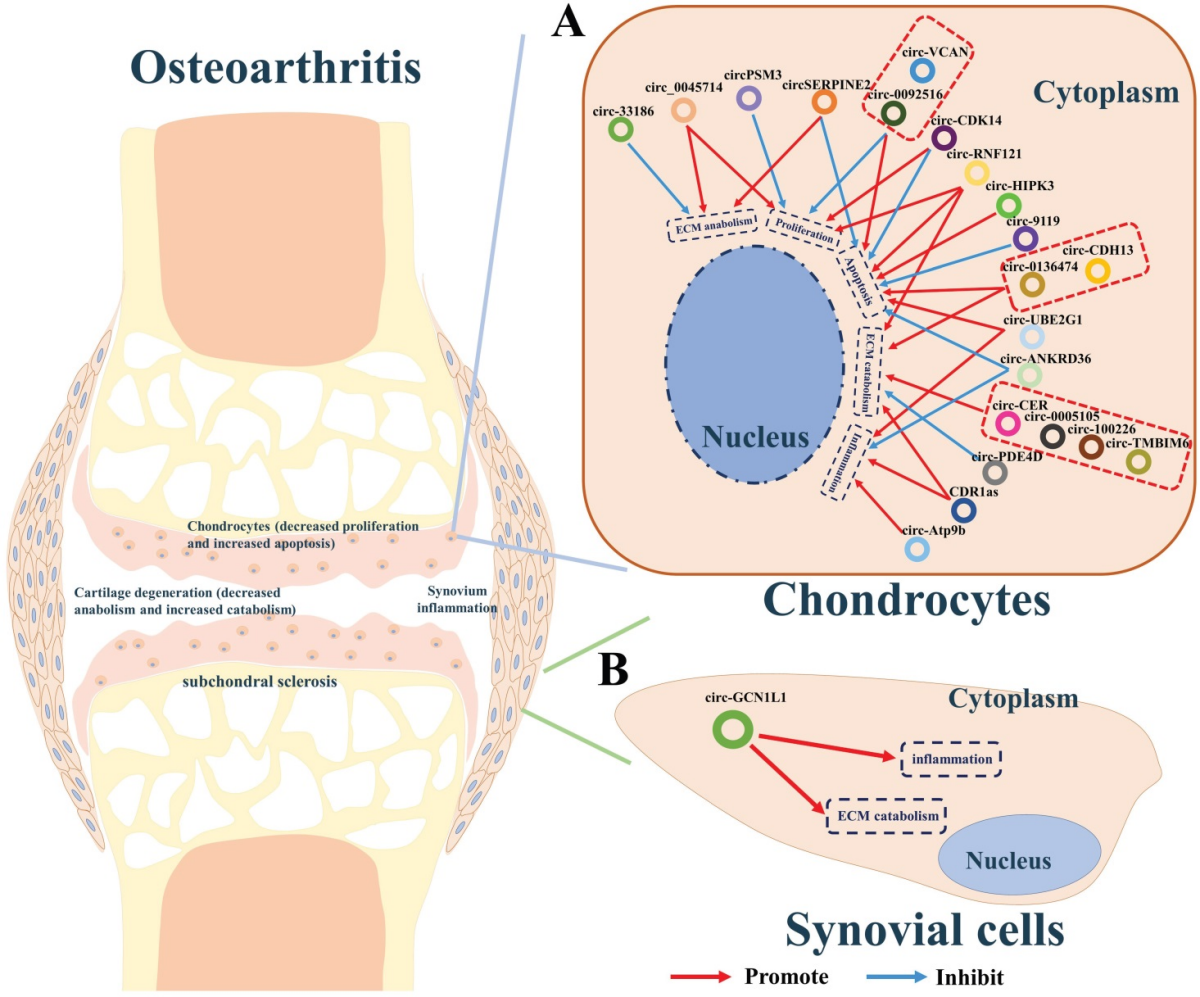
** A summary diagram of circRNAs in regulating osteoarthritis. (A)** CircRNAs CDR1as, circ-0005105, circ-33186, circ-0136474, circ-100226, circ-CER, circ-PSM3, circ-Atp9b, circ-UBE2G1, circ-0092516, circ-CDH13, circ-TMBIM6, circ-RNF121, circ-VCAN, and circ-HIPK3 could significantly promote the progress of osteoarthritis by stimulating ECM catabolism, apoptosis, inflammation, but suppressing cell proliferation and ECM anabolism of chondrocytes thereby aggravating the progress of osteoarthritis. While circ-SERPINE2, circ-CDK14, circ-ANKRD36, circ-PDE4D, circ-0045714, and circ-9119 could inhibit the progress of osteogenesis by stimulating cell proliferation, ECM anabolism, but suppressing ECM catabolism, apoptosis, inflammation of chondrocytes thereby hindering the progress of osteoarthritis. **(B)** CircGCN1L1 could promote synoviocyte proliferation and chondrocyte apoptosis in osteoarthritis thereby aggravating the progress of osteoarthritis.

Increasing evidence indicates that some circRNAs plays pivotal roles in the development of osteoarthritis. We summarized the circRNAs dysregulated in articular cartilage and synovium** (Table 2)**. Furthermore, the downstream pathways caused by these dysregulated circRNAs finally result in inflammation, the imbalance between anabolism and catabolism of ECM, inhibition of cells proliferation, or apoptosis. Studies have shown that CDR1as 49, circ-0005105 50, circ-33186 51, circ-0136474 52, circ-100226 32, circ-CER 53, circ-PSM3 54, circ-Atp9b 55, circ-UBE2G1 56, circ-0092516 57, circ-CDH13 58, circ-TMBIM6 59, circ-RNF121 60, circ-VCAN 61, and circ-HIPK3 62, which were found over-expressed in cartilage of osteoarthritis, could significantly promote the expression of osteoarthritis-related genes including MMP13, PTEN, FGF2, NAMPT, TNFα, TLR4, HIFα, MYD88, and SOX8, thereby accelerating the progress osteoarthritis. While circ-SERPINE2 63, 64, circ-CDK14 65, circ-ANKRD36 66, circ-PDE4D 67 circ-0045714 68, and circ-9119 69 were found down-expressed and have positive effects on alleviating the progress of osteogenesis **(Figure 2A)**.

Additionally, synovitis is the main cause of joint pain in osteoarthritis 70. CircGCN1L1 was found upregulated in the synovium and play its roles by promoting synoviocyte proliferation and chondrocyte apoptosis in osteoarthritis. Silencing of circGCN1L1 attenuates the loss of condylar cartilage and subchondral bone via the circGCN1L1-miR-330-3p-TNF axis **(Figure 2B)** 71.

These osteoarthritis-related circRNAs may function as novel therapeutic targets for the treatment and prevention of osteoarthritis. Future work exploring the upstream regulation of aberrantly expressed circRNAs in osteoarthritis, as well as the molecular details of circRNA-protein interactions in osteoarthritis, will be required to further understand the therapeutic potential of osteoarthritis -related circRNAs.

## CircRNAs and ONFH

Osteonecrosis is a class of orthopedic diseases that is caused by the interruption of blood flow, affecting over 20 million people worldwide 72-74. Due to its anatomical structure, the femoral head is particularly likely to undergo osteonecrosis 75. Common risk factors of ONFH include excessive use of steroids or alcohol, trauma, or sickle cell anemia 76, all of which reduce blood supply to the femoral head, thus resulting in bone necrosis and alteration 77. Important to the prevention of bone necrosis is bone regeneration via the differentiation and proliferation of bone marrow stromal cells (BMSCs) 78. However, an imbalance of osteogenesis and adipogenesis of BMSCs has been observed to disrupt bone remodeling. Studies have revealed that circRNAs are dysregulated during osteogenesis. Here, we review circRNAs that have been shown to play critical roles in regulating osteogenesis and adipogenesis, to shed light on a regulatory mechanism for steroid-induced osteonecrosis of the femoral head (SONFH).

**Figure 3:**
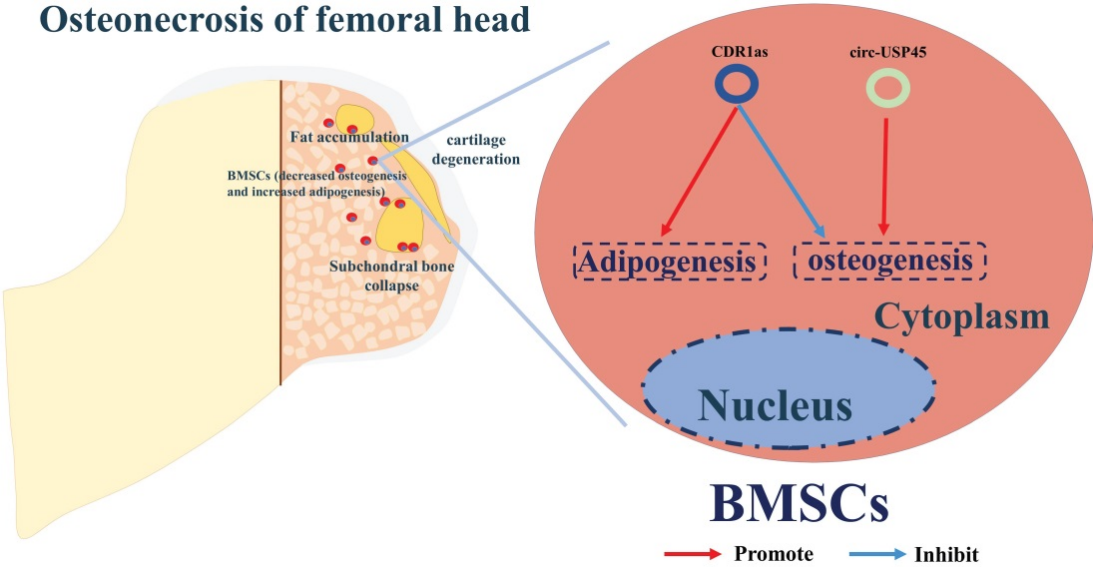
** A summary diagram of circRNAs in regulating ONFH.** CircUSP45 and CDR1as could promote the progress of ONFH by inhibiting osteogenesis but enhancing adipogenesis in BMSCs, thereby aggravating the progress of ONFH.

The correlation between circRNAs and ONFH is still in its infancy. To date, studies of the ONFH-related circRNAs have mainly highlighted their roles in osteogenesis or adipogenesis of BMSCs, the imbalance of which has a critical role in the progression of ONFH **(Table 2)** 36, 79, 80. By screening the circRNA expression profiles in BMSCs from patients with SONFH, and using bioinformatics and functional characterization assays, CDR1as was found up-regulated in SONFH-BMSCs, and thus could play a critical role in osteogenic/adipogenic differentiation disorders via the miR-7-5p/WNT5B axis of regulation **(Figure 3)** 36. Kuang et al. identified circUSP45 as an upregulated circRNA in BMSCs isolated from SONFH patients 80. RNA pull-down and dual luciferase reporter assays were performed to confirm that circUSP45 mainly localizes in the cytoplasm and directly interacts with miR-127-5p. Further experiments verified that circUSP45 upregulates the expression of PTEN and inhibits AKT pathway by sponging miR-127-5p, thereby suppressing the expression of osteogenic genes, such as bone morphogenetic protein-2 (BMP2) and runt-related transcription factor 2 (RUNX2). Additionally, the anti-bone metabolism function of circUSP45 was verified *in vivo* by a SONFH rat model.

Therefore, silencing CDR1as and circUSP45 may promote bone metabolism and improve bone mass in SONFH progression. However, future work is required to understand more mechanistic details of regulatory circRNAs and their roles in the pathogenesis of ONFH **(Figure 3)**.

## CircRNAs and osteoporosis

Osteoporosis is a systemic and metabolic skeletal disorder affecting with more than 200 million individuals affected worldwide 81, 82. Osteoblastic bone formation and osteoclastic bone resorption dynamically maintain bone homeostasis 83. Osteoporosis is typically characterized by decreased osteoblastic activity and increased osteoclastic activity 84. During osteoporosis, BMSCs, which are precursors to osteoblasts, have been shown to have a lower osteogenic differentiation potential 85. Regulatory mechanisms underlying osteoporosis are complex and involve a number of pathways, including circRNA mediation pathway.

### Diagnostic circRNAs of osteoporosis

Here, we summarized circRNAs that are abnormally expressed in human bodily fluids from osteoporosis patients. These circRNAs may be promising candidates for clinical diagnosis of osteoporosis. In particular, circ_0002060 was shown by Huang et al. via microarray and bioinformatic analyses, to be upregulated in clinical samples, suggesting its use as a potential biomarker (**Table 1**) 86, 87.

### Regulatory circRNAs of osteoporosis

**Figure 4:**
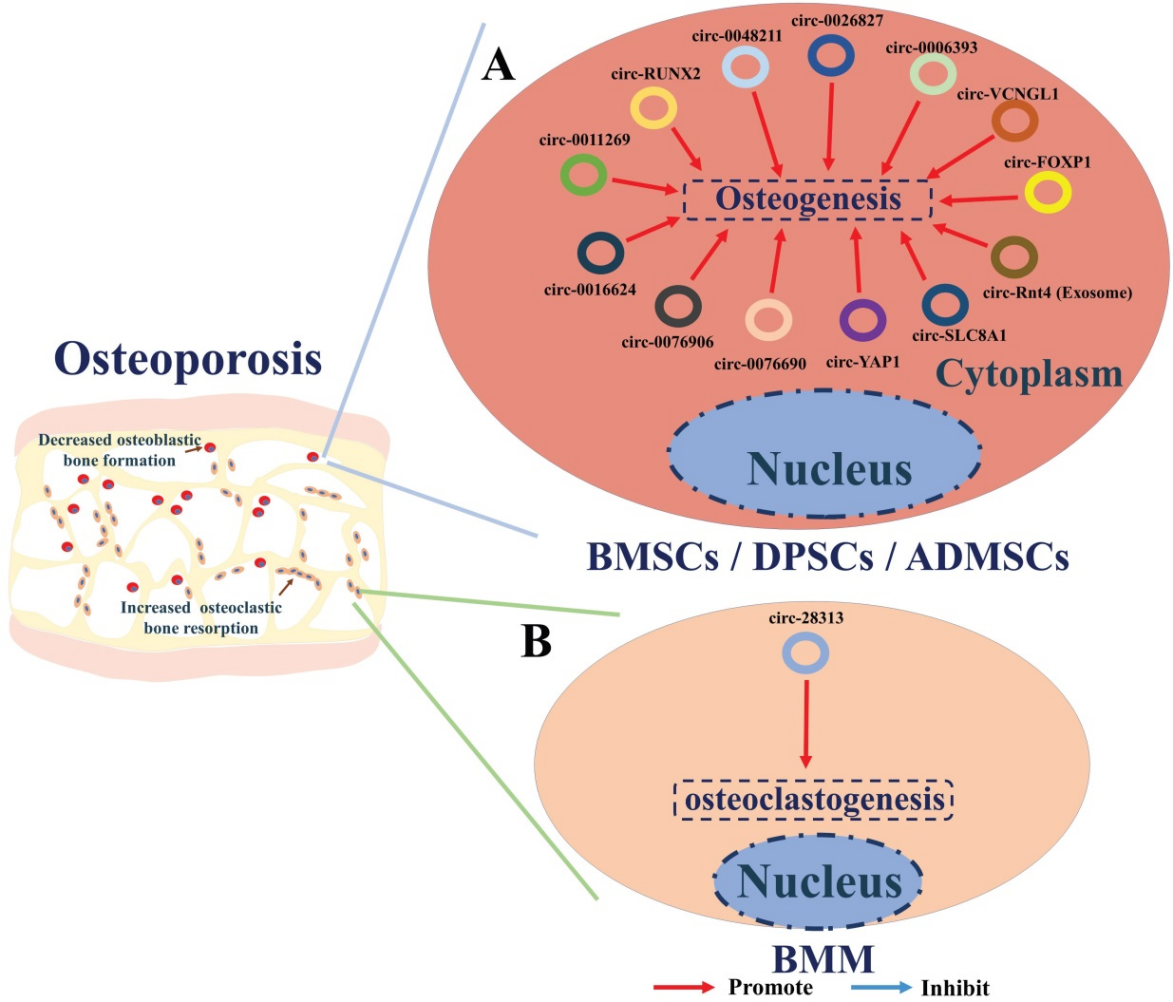
** A summary diagram of circRNAs in regulating osteoporosis. (A)** CircRNAs circ-RUNX2, circ-VANGL1, circ-0011269, circ-0076906, circRNA-0016624, circ-0006393, circRNA-0048211, circ-SLC8A1, circ-YAP1, circ-0076690, and circ-Rtn4 (exosomes) could promote the osteogenesis of BMSCs thereby hindering the progress of osteoporosis. Circ_0026827 could promote the osteogenesis of human dental pulp stem cells (DPSCs) thereby hindering the progress of osteoporosis. CircFOXP1 could promote the osteogenesis of adipose-derived mesenchymal stem cells (ADMSCs) thereby hindering the progress of osteoporosis. **(B)** While circ-28313 could promote the osteoclastogenesis of bone marrow monocyte /macrophage (BMM) thereby aggravating the progress of osteoporosis.

CircRNAs participate in the regulation of osteoporosis via several ways **(Table 2)**. Recent researches have screened the circRNAs expression profile in bone and serum of patients with osteoporosis. The majority of osteoporosis -related circRNAs are involved in regulating osteogenesis by sponging miRNAs and consequently regulating the expression or activity of downstream osteogenesis genes. Circ-RUNX2 88, circ-VANGL1 89, circ-0011269 90, circ-0076906 91, circRNA-0016624 92, circ-0006393 93, circRNA-0048211 94, circ-SLC8A1 95, circ-YAP1 96 and circ-0076690 97 are found down-regulated in osteoporosis and could enhance osteogenesis of BMSCs by sponging miRNAs and subsequently upregulating the expression and activities of osteogenetic genes (such as RUNX2, BMP2, OPN, OCN, OGN, FOXO1, APAK2 or ALP) (**Figure 4A**). Exosomes of BMSCs (BMSCs-Exos) have also been shown to function in bone regeneration 98-100, while exosomes-derived from circ-Rtn4 have been shown to promote osteogenesis by targeting miR-146a 101. Studies performed by Cao et al. revealed that the expression of miR-146a is positively correlated with TNF-α, a cytokine that serves as a key regulator of osteoporosis pathology. Additionally, they showed that circ-Rtn4 attenuates TNF-α-induced cytotoxicity and apoptosis in MC3T3-E1 cells by acting as a sponge for miR-146a, implicating Rtn4-Exos as a promising therapeutic candidate for osteoporosis **(Figure 4A)** 101.

Some studies have also shown the regulatory mechanism of circRNAs in other types of stem cells. The effect of circ_0026827 on human dental pulp stem cells (DPSCs) during osteogenesis has been examined by Ji et al. 102 to seek novel therapeutics for osteoporosis. These studies showed that circ_0026827 function as a sponge of miR-188-3p to promote osteoblastic differentiation of DPSCs via the upregulation of Beclin-1-mediated autophagy and RUNX1 signaling pathways. CircFOXP1 were found to play critical roles in promoting osteogenic differentiation of adipose-derived mesenchymal stem cells (ADMSCs) in osteoporosis by targeting miR-33a-5p. *In vivo* and *in vitro* evidence indicates circFOXP1 could enhance the expression of FOXP1, thereby promote the osteogenic differentiation of ADMSCs and bone formation in osteoporosis **(Figure 4A)** 103.

To date, circRNA_28313 is the only circRNA that has been shown to be a regulator of osteoclast resorption. Recent studies by Chen et al. 104 have examined mechanisms, involving circRNA regulation, of excessive bone resorption caused by osteoclasts during their differentiation. The authors used RANKL+CSF1-treated bone marrow monocyte/macrophage (BMM) cells to mimic osteoclasts during the progression of osteoporosis. Their results showed that circRNA_28313 is dramatically overexpressed upon RANKL+CSF1 stimulation, while miR-195a is down-expressed. Further bioinformatics and experimental validation both* in vitro* and *in vivo* showed that circRNA_28313 interacts with miR-195a and consequently regulates the colony-stimulating factor (CSF1) gene. High expression level of CSF1 in turn, promotes the expression of downstream proteins such as PU.1 (a factor related to osteoclast differentiation), nuclear factor of activated T cells c1 (NF-ATc1), tartrate-resistant acid phosphatase (TRAP), and Cathepsin K (CTSK), thereby enhancing osteoclast-induced bone absorption **(Figure 4B)**.

Thus, circRNAs can serve as powerful therapeutic candidates to promote bone regeneration and thus reverse the progression of osteoporosis. Moreover, a deeper understanding of these circRNAs can shed light on the mechanisms underlying regulation of circRNAs in bone metabolism.

## CircRNAs and rheumatoid arthritis

Rheumatoid arthritis (RA) is a common chronic autoimmune disease characterized by inflammatory destruction that can cause serious cartilage and bone damage, affecting approximately 1% of the population worldwide 30. Several non-coding RNAs have been identified as regulators of RA via different pathways, but the exact mechanisms underlying the role of circRNAs in RA remain to be understood.

### Diagnostic circRNAs of rheumatoid arthritis

Early diagnosis is critical to optimal therapeutic success for rheumatoid arthritis (RA). Existing studies have identified a several circRNAs that could serve as diagnostic biomarkers of RA, partially due to their increased stability in plasma, serum, or other biofluids 105. Here, we summarizes potential biomarkers identified by various studies **(Table 1)** 106-111.

### Regulatory circRNAs of rheumatoid arthritis

The main pathological features of RA are autoimmune response and inflammation 112. Recently, a number of circRNAs have been implicated in RA pathogenesis **(Table 2)**, but their function and hidden molecular mechanism in immune and inflammation regulation still remains little known 30.

**Figure 5:**
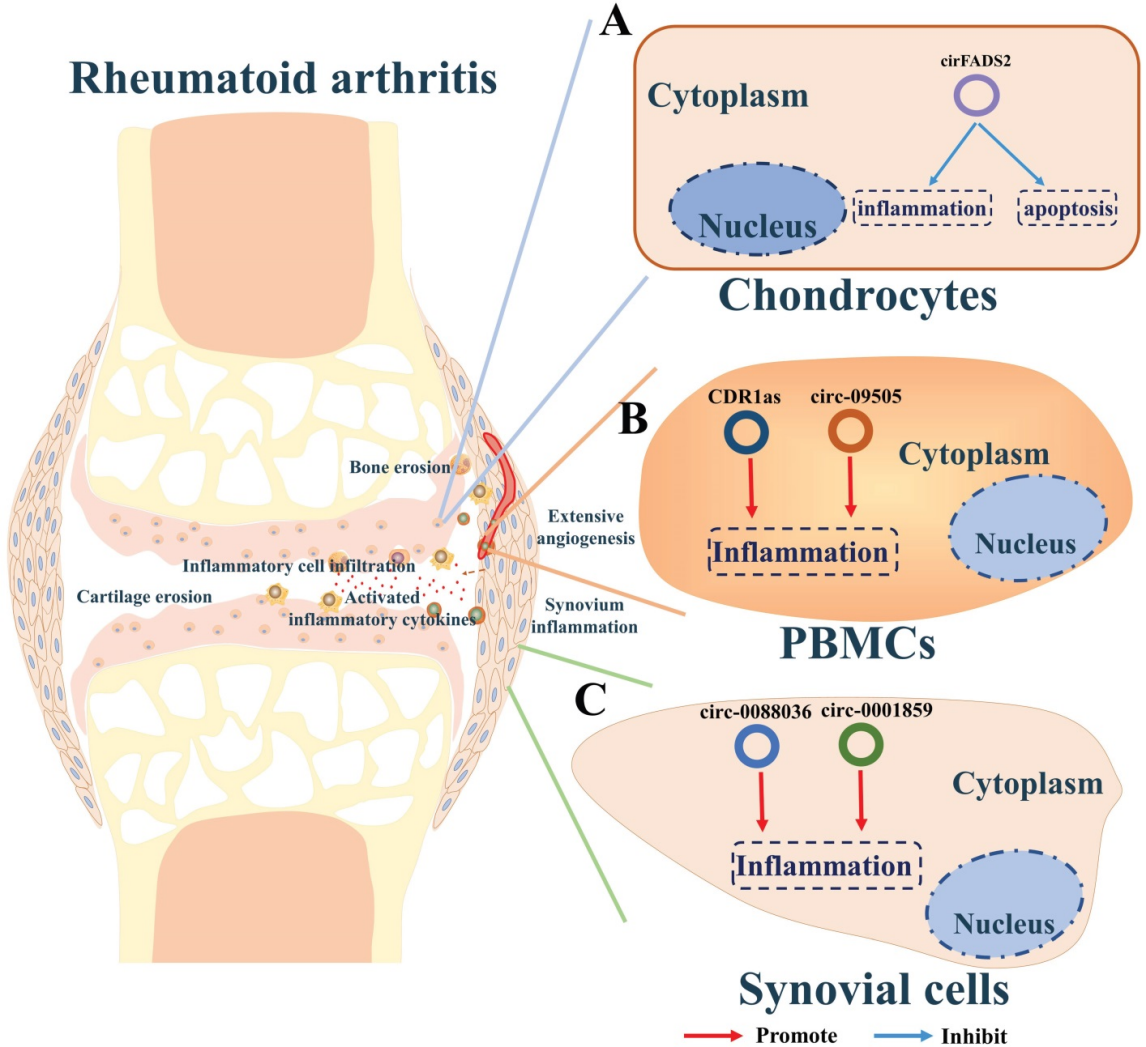
** A summary diagram of circRNAs in regulating RA. (A)** CircRNA circFADS2 could protect the chondrocytes from apoptosis and inflammation thereby hindering the progress of rheumatoid arthritis. **(B)** CDR1as and circ-09505 could enhance the inflammation of PBMCs thereby promoting the progress of rheumatoid arthritis.** (C)** Circ-0001859 and circ-0088036 could promote the inflammation of synovium in rheumatoid arthritis.

Given that the phosphatidylinositol-3-kinase/AKT/mTOR (PI3K/AKT/mTOR) signaling pathway plays a crucial role in cellular proliferation and inflammatory responses 113, 114, circFADS2 and ciRS-7 have been implicated in the initiation and progression of RA by regulating these pathways. CiRS-7 promotes the inflammation of PBMCs by sponging miR-7 to upregulate mTOR while circFADS2 protects LPS-treated chondrocytes (RA model cells) from apoptosis by mediating mTOR expression via sponging miR-498** (Figure 5A and B)** 115-117. CircRNA_09505 is an up-regulated circRNA which can promote AKT1 expression via miR-6089/IκBα/NFκB signaling pathway in macrophages, thereby aggravating inflammation and joint damage in RA** (Figure 5B)** 118.

Joint swelling and pain reflect synovial membrane inflammation in RA, resulting from the immune activation and infiltration of leucocyte. Li and colleagues examined the expression of circRNAs in synovial tissues and screened out circ-0001859 as a critical RA-related circRNA 119. Their further studies indicated that circ-0001859 could up-regulate ATF2 via sponging miR-204/211 in synovial sarcoma cells (SW982 cells). Silencing circ_0001859 reduced the hyper inflammatory activity in the synovial tissue and alleviates the pathogenesis of RA **(Figure 5C)**. Circ-0088036 was found to be aberrantly upregulated in fibroblast-like synoviocytes (FLSs) in RA 120. Circ-0088036 could promote the proliferation and migration of RA-FLSs via miR-140-3p/SIRT1 axis, subsequently promoting RA progression **(Figure 5C)**.

The studies described above highlight the effects of circRNAs on RA, but a great deal remains to be understood. Future studies are required to further understand the underlying mechanisms of regulation of RA progression by circRNAs.

## Conclusions and future perspective

CircRNA is a class of noncoding RNA molecules with a closed loop structure formed by covalent bonds, which can protect them from degradation by most RNases 121. Emerging studies have exploited highly-accurate circRNA biomarkers in human body fluids for diagnosis and prognosis of some diseases, such as cancer and cardiovascular diseases 122, 123. In recent years, studies have also revealed the potential value of circRNAs in clinical treatment of various diseases, including skeletal and chondral disorders. In this review, we have reviewed the regulatory role of circRNA in skeletal and chondral disorders and summarized findings of several circRNAs that play critical roles in the process of osteoarthritis, ONFH, osteoporosis, or RA. These findings may provide further insight into the mechanistic details of the role of circRNAs in bone or cartilage metabolism, and highlight potential targets for the clinical treatment of orthopedic diseases. Nevertheless, the molecular functions of circRNAs in skeletal and chondral disorders still remain largely enigmatic. Their regulatory mechanism in skeletal and chondral disorders by regulating transcription, binding protein, and encoding protein or peptides still remains unknown, which is worth to be further explored in later research on skeletal and chondral disorders.

Despite recent discoveries summarized above, many challenges remain to be overcome. The functional verification of circRNAs implicated in skeletal and chondral disorders, has been mostly *in vitro*. *In vivo* studies are a greater challenge due to the lack of stable and specific delivery vehicles. Recent studies have discovered some biological vehicle that can be loaded with circRNA (or si-circRNA) for *in vivo* therapy, including extracellular vesicles (EVs) and adeno-associated viral (AAV) vectors. EVs are a heterogeneous group of lipid bilayer-enclosed nanosized vesicles releasing from various types of cells 124. Many studies have highlighted the application of EVs in transporting circRNA to specific tissues by modification 125, 126. AAV is a non-pathogenic member of the Parvovirus family which can deliver circRNA producing transgenes 127. Studies have also revealed the potential value of recombinant AAV in targeting therapy via genetic modification 128. These advances may shed new lights on the application of circRNA in the treatment of skeletal and chondral disorders. We believe that an in-depth understanding and correct application of circRNAs in clinical practice will make a giant progress in the treatment of skeletal and chondral disorders in the near future.

## Figures and Tables

**Table 1:** CircRNAs as potential biomarkers of skeletal and chondral disorders.

Diseases	CircRNAs	Samples	Expression	Methods	Ref.
OA	Hsa_circ_0104873	synovial fluid	up	CircRNA-array	46
OA	Hsa_circ_ 0104595	synovial fluid	up	CircRNA-array	46
OA	Hsa_circ_0101251	synovial fluid	up	CircRNA-array	46
OA	Hsa_circ_0032131	peripheral blood	up	CircRNA-array	47
OP	Hsa_circ_0002060	serum and plasma	up	CircRNA-array	86
OP	Hsa_circ_0001445	plasma	down	qRT-PCR	87
RA	Hsa_circ_0044235	peripheral blood	down	qRT-PCR	106
RA	Hsa_circ_102594	PBMSs	down	CircRNA-array	107
RA	Hsa_circ_104194	PBMSs	up	CircRNA-array	107
RA	Hsa_circ_104593	PBMSs	up	CircRNA-array	107
RA	Hsa_circ_103334	PBMSs	up	CircRNA-array	107
RA	Hsa_circ_101407	PBMSs	up	CircRNA-array	107
RA	Has_circ_0008360	PBMSs	down	RNA-seq	108
RA	Has_circ_0001200,	PBMSs	up	RNA-seq	108
RA	Has_circ_0001566	PBMSs	up	RNA-seq	108
RA	Has_circ_0003972	PBMSs	up	RNA-seq	108
RA	Hsa_circ_0002715	peripheral blood	up	qRT-PCR	109
RA	Hsa_circ_0035197	peripheral blood	up	qRT-PCR	109
RA	Hsa_circ_0000175	PBMSs	down	qRT-PCR	110
RA	Hsa_circ_0008410	PBMSs	up	qRT-PCR	110
RA	circRNA_104871	PBMSs	up	CircRNA-array	111
RA	circRNA_003524	PBMSs	up	CircRNA-array	111
RA	circRNA_101873	PBMSs	up	CircRNA-array	111
RA	circRNA_103047	PBMSs	up	CircRNA-array	111

OA: osteoarthritis; OP: osteoporosis; RA: rheumatoid arthritis.

**Table 2:** Regulatory circRNAs and their roles in skeletal and chondral disorders.

Disease	CircRNA	Tissue	Expression	miRNA/gene	Roles	Ref.
OA	circ-100226	cartilage	up	miR-875/TNFα	ECM catabolism ↑	32
OA	CDR1as	cartilage	up	miR-641/FGF2	inflammation ↑, ECM catabolism ↑	49
OA	circ-0005105	cartilage	up	miR-26a/NAMPT	ECM catabolism ↑	50
OA	circ-33186	cartilage	up	miR-217-5p/MMP13	cell proliferation ↓	51
OA	circ-0136474	cartilage	up	miR-217-5p/MMP13	ECM catabolism ↑, apoptosis ↑	52
OA	circ-CER	cartilage	up	miR-136/MMP13	ECM catabolism ↑	53
OA	circ-PSM3	cartilage	up	miR-296-5p	cell proliferation ↓	54
OA	circ-Atp9b	cartilage	up	miR-138-5p/TLR4	inflammation ↑	55
OA	circ-UBE2G1	cartilage	up	miR-373/HIFα	inflammation ↑, apoptosis ↑	56
OA	circ-0092516	cartilage	up	miR-337-3p/PTEN	apoptosis ↑, cell proliferation ↓	57
OA	circ-CDH13	cartilage	up	miR-296-3p/PTEN	ECM catabolism ↑, apoptosis ↑	58
OA	circ-TMBIM6	cartilage	up	miR-27a/MMP13	ECM catabolism ↑	59
OA	circ-RNF121	cartilage	up	miR-665/MYD88	ECM catabolism ↑, apoptosis ↑, cell proliferation ↓	60
OA	circ-VCAN	cartilage	up	NF-κB	apoptosis ↑, cell proliferation ↓	61
OA	circ-HIPK3	cartilage	up	miR-124/SOX8	apoptosis ↑	62
OA	circ-SERPINE2	cartilage	down	miR-1271-5p/ERG	ECM anabolism ↑	63
		cartilage	down	miR-495/ TGFBR2	apoptosis ↓	64
OA	circ-CDK14	cartilage	down	miR-125a-5p/SMAD2	apoptosis ↓, cell proliferation ↑	65
OA	circ-ANKRD36	cartilage	down	miR-599/CAS21	inflammation ↓, apoptosis ↓	66
OA	circ-PDE4D	cartilage	down	miR-103a-3p/FGF18	ECM catabolism ↓	67
OA	circ-0045714	cartilage	down	miR-1936/IGF1R	cell proliferation ↑, ECM anabolism ↑	68
OA	circ-9119	cartilage	down	miR-127-5p/PTEN	apoptosis ↓	69
OA	circ-GCN1L1	synovium	up	miR-330-3p/TNFα	inflammation ↑, ECM catabolism ↑	71
ONFH	CDR1as	BMSCs	up	miR-7-5p/WNT5B	osteogenesis ↓, adipogenesis ↑	36
ONFH	circ-USP45	BMSCs	up	miR-127-5p/PTEN	osteogenesis ↓	80
OP	circ-RUNX2	bone	down	miR-203/RUNX2	osteogenesis ↑	88
OP	circ-VCNGL1	serum	down	miR-217-5p/RUNX2	osteogenesis ↑	89
OP	circ-0011269	serum	down	miR-122/RUNX2	osteogenesis ↑	90
OP	circ-0076906	serum/bone	down	miR-1305/OGN	osteogenesis ↑	91
OP	circ-0016624	serum	down	miR-98/BMP2	osteogenesis ↑	92
OP	circ-0006393	BMSCs	down	miR-145-5p/FOXO1	osteogenesis ↑	93
OP	circ-0048211	BMSCs	down	miR-93-5p/BMP2	osteogenesis ↑	94
OP	circ-SLC8A1	Bone	down	miR-516b-5p/APAK2	osteogenesis ↑	95
OP	circ-YAP1	BMSCs	down	miR-376b-3p/YAP1	osteogenesis ↑	96
OP	circ-0076690	serum	down	miR-152/RUNX2	osteogenesis ↑	97
OP	circ-RTN4	exosome	down	miR-1446a/TNFα	osteogenesis ↑	101
OP	circ-0026827	DPSCs	down	miR-188-3p/RUNX1	osteogenesis ↑	102
OP	circ-FOXP1	ADMSCs	down	miR-330-5P/FOXP1	osteogenesis ↑	103
OP	circ-28313	BMM	up	miR-195a/ CSF1	osteoclastogenesis ↑	104
RA	CDR1as	PBMCs	up	miR-7-5p/ mTOR	inflammation ↑	116
RA	circ-FADS2	cartilage	down	miR-498/mTOR	apoptosis↓, inflammation ↓	117
RA	circ-09505	PBMCs	up	miR-6089/AKT1	inflammation ↑	118
RA	circ-0001859	synovium	up	miR-204/211/ATF2	inflammation ↑	119
RA	circ-0088036	synovium	up	miR-140-3p/SIRT1	inflammation ↑	120

OA: osteoarthritis; ONFH: osteonecrosis of the femoral head; OP: osteoporosis; RA: rheumatoid arthritis.

## Abbreviations

circRNAs: circular RNAs
ncRNAs: non-coding RNAs
EcRNAs: exonic circRNAs
ciRNAs: intronic circRNAs
EIcRNAs: exon-intron circRNAs
OA: osteoarthritis
ONFH: osteonecrosis of the femoral head
OP: osteoporosis
RA: rheumatoid arthritis
ECM: extracellular matrix
ERG: E26 transformation-specific-related gene
IL-1β: interleukin-1 beta
NAMPT: nicotinamide phosphoribosyltransferase
Col II: type II collagen
TNF-α: tumor necrosis factor
IGF1R: insulin-like growth factor 1 receptor
LPS: lipopolysaccharide
BMSCs: bone marrow stromal cells
PTEN: phosphate and tension homology deleted on chromsome ten
BMP2: bone morphogenetic protein-2
RUNX2: runt-related transcription factor 2
BMM: bone marrow monocyte/macrophage
RANKL: receptor activator of nuclear factor-κB ligand
CSF1: colony-stimulating factor
NF-ATc1: nuclear factor of activated T cells c1
TRAP: tartrate-resistant acid phosphatase
CTSK: cathepsin K
OCN: osteocalcin
OPN: osteopontin
BSP: bone sialoprotein
ALP: alkaline phosphatase
OGN: osteoglycin
DPSCs: human dental pulp stem cells
FOXO1: forkhead box o1
BMSCs-Exos: exosomes of BMSCs
PBMCs: peripheral blood mononuclear cells
ATF2: activating transcription factor 2
